# COVID-19 Information Sources and Health Behaviors During Pregnancy: Results From a Prenatal App-Embedded Survey

**DOI:** 10.2196/31774

**Published:** 2021-12-07

**Authors:** James Bohnhoff, Alexander Davis, Wändi Bruine de Bruin, Tamar Krishnamurti

**Affiliations:** 1 Division of General Pediatrics University of Pittsburgh School of Medicine Pittsburgh, PA United States; 2 Department of Engineering and Public Policy Carnegie Mellon University Pittsburgh, PA United States; 3 Sol Price School of Public Policy University of Southern California Los Angeles, CA United States; 4 Schaeffer Center for Health Policy and Economics University of Southern California Los Angeles, CA United States; 5 Division of General Internal Medicine University of Pittsburgh School of Medicine Pittsburgh, PA United States

**Keywords:** COVID-19, health behavior, health behaviour, pregnancy, obstetrics, perinatal, preventive, preventative, mHealth, risk, information source, medical literacy, media literacy, information literacy, protection, protective, harm, women, engagement, online health information, behavior, information-seeking, critical appraisal, communication

## Abstract

**Background:**

Pregnancy is a time of heightened COVID-19 risk. Pregnant individuals’ choice of specific protective health behaviors during pregnancy may be affected by information sources.

**Objective:**

This study examined the association between COVID-19 information sources and engagement in protective health behaviors among a pregnant population in a large academic medical system.

**Methods:**

Pregnant patients completed an app-based questionnaire about their sources of COVID-19 information and engagement in protective health behaviors. The voluntary questionnaire was made available to patients using a pregnancy app as part of their routine prenatal care between April 21 and November 27, 2020.

**Results:**

In total, 637 pregnant responders routinely accessed a median of 5 sources for COVID-19 information. The most cited source (79%) was the Centers for Disease Control and Prevention (CDC). Self-reporting evidence-based protective actions was relatively common, although 14% self-reported potentially harmful behaviors to avoid COVID-19 infection. The CDC and other sources were positively associated with engaging in protective behaviors while others (eg, US president Donald Trump) were negatively associated with protective behaviors. Participation in protective behaviors was not associated with refraining from potentially harmful behaviors (*P*=.93). Moreover, participation in protective behaviors decreased (*P*=.03) and participation in potentially harmful actions increased (*P*=.001) over the course of the pandemic.

**Conclusions:**

Pregnant patients were highly engaged in COVID-19–related information-seeking and health behaviors. Clear, targeted, and regular communication from commonly accessed health organizations about which actions may be harmful, in addition to which actions offer protection, may offer needed support to the pregnant population.

## Introduction

Pregnant people are at higher risk for severe COVID-19 illness and adverse pregnancy outcomes such as hypertensive disorders, preterm birth, and cesarean delivery [1,2]. However, the risk of vertical transmission of COVID-19 is still being studied [3], and data on efficacy and safety of COVID-19 vaccination for pregnant women lag behind those for other populations [4]; furthermore, recommendations on appropriate health action differ by information source, including conflicting advice by professional health organizations [5,6]. Thus, pregnant people are faced with heightened risk and less certain information when seeking knowledge of appropriate COVID-19–related health choices. Even if highly motivated to engage in positive health behaviors, pregnant people have not always known what actions would offer them appropriate protection, with many doubting the benefits of protective behaviors such as vaccination [7,8].

In the broader US population, adoption of protective behaviors continues to be uneven, despite a growing scientific consensus on effective protective behaviors to decrease the transmission and contraction of COVID-19 [9,10]. Individuals’ information sources may be an important determinant of health beliefs, behaviors, and the acceptance of health guidance [11]. Use of news sources such as the Centers for Disease Control and Prevention (CDC) has been associated with COVID-19 knowledge [12] and protective action such as social distancing [13]. People have also sought COVID-19 information from other sources including social media [12], where evidence-based guidelines were often drowned out by misinformation [14] and “echo chambers” [15]. The sources that pregnant people access may, therefore, inform their willingness to implement protective behaviors.

Here, we used data collected through a pregnancy health tracking app with the aim of examining the relationship between the sources from which pregnant people seek COVID-19–related health information and their engagement in protective health behaviors. Specifically, we examined whether the information source chosen for learning about COVID-19 was associated with (1) engagement in evidence-based protective health behaviors, such as hand-washing and social distancing, and (2) potentially harmful behaviors that have been perpetuated through misinformation, such as personal use of UV radiation to treat or prevent infection [16,17] while accounting for demographic and clinical covariates. We also examined (3) whether participation in evidence-based behaviors was associated with refraining from harmful actions. We hypothesized that pregnant individuals’ COVID-19 information sources are associated with their effective and potentially harmful behaviors, and that higher levels of effective health behaviors would be associated with lower levels of harmful behaviors.

## Methods

### Data Collection Tool

Providers at the University of Pittsburgh Medical Center health system prescribed the MyHealthyPregnancy (MHP) app (iOS version 1.4.7, Android version 1.8) to pregnant patients at their first prenatal appointment as part of routine prenatal care. All content was developed in conjunction with a clinical education team employed by the health care system. MHP applies machine learning algorithms to patient-entered data to model an individual patient’s likelihood of adverse pregnancy events. The app offers relevant resources (eg, local health services) or actions (eg, prompts to call their provider), depending on the information that is entered into the app, as well as notifying their provider if critical health risks are documented. From April 2020, MHP added questions about COVID-19 symptoms (COVID-19 screening tool), responding to symptom reports with care-seeking guidance, and a separate COVID-19 behaviors questionnaire that included questions about COVID-19 information sources and engagement in specific protective behaviors. App users were then also offered some additional education about appropriate protective behaviors. Surveys were checked against the Checklist for Reporting the Results of E-Surveys (CHERRIES), focusing on items relevant to an app-based survey [18].

The internal protocol for prescribing MHP was to send a weblink to the patient’s phone. App users electronically consented to share identifiable data with their health care provider and anonymized aggregate data for research. Participants did not receive any financial compensation for app use.

During the patient’s first use of MHP (onboarding), they were prompted with 26 multiple-choice questions, over 4 screens of questioning, which included questions on demographics and pregnancy history. During the study period (April 21 to November 27, 2020), participants were invited via an SMS text message and in-app notification to voluntarily complete the COVID-19 screening tool (4 questions) and COVID-19 behaviors questionnaire (8 questions). The app’s “Learning Center” was then updated for all app users, regardless of use of the screening tool or participation in the COVID-19 behaviors questionnaire. The COVID-19 screening tool remained available for use at any time.

### COVID-19–Related Information Sources and Protective Actions

As part of the COVID-19 behaviors questionnaire, participants indicated where they received their coronavirus-related information from a list of choices composed of government entities, media sources, the internet and social media, and personal contacts, with the option to list additional sources through free text. Participants were also asked to select actions that they had taken in the last month to keep themselves safe from COVID-19. These actions are enumerated in the Results section. The research team reviewed the first behaviors questionnaire completed by each participant. We categorized actions on their evidence base and potential health risk. Three actions, including (1) avoiding public spaces, gatherings, or crowds, (2) washing hands with soap or using hand sanitizer several times per day, and (3) wearing a face mask, were categorized as “most effective” in accordance with CDC recommendations [19]. Other actions, such as “cancel[ing] or postpon[ing] air travel for work” were categorized as “protective actions” on the basis that they were of known benefit but overlapped with the “most effective” actions or were not applicable to every individual. Actions related to scheduling or canceling medical appointments were categorized as indeterminate. Actions identified by the CDC and the World Health Organization (WHO) as commonly reported misconceptions were categorized as “Other unnecessary or ineffective” for preventing COVID-19 (eg, “Stockpiled food or water”) or “Ineffective and potentially harmful” (eg, “Used antibiotics”) [20,21]. For each participant, we recorded the number of “most effective” actions selected as well as the selection of any “potentially harmful” actions, focusing on these 2 categories as the most likely to be of interest to organizations hoping to reduce COVID-19 spread and prevent harm. This categorization system was developed during analysis in late 2020 but attempted to describe recommendations which had been relatively consistent throughout the pandemic. In particular, wearing a face mask, though initially discouraged by the CDC, was recommended as a voluntary, protective health measure beginning early April 2020 [22].

### Other Health Information

Respondents were designated as having a high-risk pregnancy history if they reported any of the following at baseline: use of in vitro fertilization or ovulation-inducing medications, prior pregnancy loss, prior premature birth (<37 weeks) or newborn with an extended hospital stay, prior premature rupture of membranes, or diagnosis of autoimmune disease, hypertension, chronic kidney disease, or diabetes. Respondents were designated as having COVID-19–relevant symptoms during the time of survey response if they reported current fever, cough, or shortness of breath in the COVID-19 screening tool. They were also asked, “Are you experiencing financial or other personal difficulties as a result of this pandemic?”

### Statistical Power

Most of our analyses are comparisons of proportions or odds ratios of respondents reporting protective or harmful actions depending on their reported information sources. With 637 respondents, we detected a statistical difference of 10 percentage points (0.6 vs 0.5) with 80% power and a Cronbach α of .05 using a 2-sample test of proportions.

### Analysis

All analyses were performed using STATA (version 15.1; StataCorp, LLC). Missing data were imputed on the basis of median and mode responses. To test the association between information sources and health behaviors, we performed 2 regression analyses. First, we used linear regression analysis to assess the association between the use of individual sources and the number of “most effective” protective actions engaged in, also factoring in the model demographics (age, race, education, number of children, and COVID-19–related distress), health characteristics (high-risk pregnancy history and COVID-19 symptoms), and survey date, which we included to measure population-level changes over the course of the pandemic. Second, we performed logistic regression analysis to test the association between the use of individual information sources and engagement in any “potentially harmful” actions. Finally, we tested the association between engagement in any “potentially harmful” factors and number of “most effective” actions undertaken using logistic regression analysis.

**Ethics Approval and Consent to Participate**

The health care system’s quality improvement review board approved the research. Informed consent was obtained from all study participants.

## Results

### Results Overview

In total, 637 women (22% of the 2906 active app users during the study period) completed the app-based COVID-19 survey, at a median gestational age of 15 weeks (IQR 10-24 weeks). Table 1 shows respondent demographics. The demographic characteristics of survey respondents were similar to those of all active app users. Respondents reported receiving information about COVID-19 from a median of 5 sources (IQR 3-7 sources). From the least to the most used source, 49 (8%) participants indicated receiving information from MSNBC, ranging to 505 (79%) participants who received information from the CDC. The most frequently cited free-text source was Dr Anthony Fauci (6 participants, 1%).

Table 2 shows the rates of respondent-reported COVID-19 protective actions, ranging from 2% (n=11, for each of “Used antibiotics” and “Used an ultraviolet disinfection lamp”) to 98% (n=626, for “Washed your hands with soap or used hand sanitizer several times per day”). Regarding the actions categorized as most effective, only 1% (n=7) of those surveyed reported practicing none of these actions and 80% (n=512) had practiced all three. In total, 89 (14%) individuals reported at least one misguided/potentially harmful action.

**Table 1:** Demographic characteristics of the surveyed women (N=637).

Characteristic		Value
Age (years), mean (SD)		30.4 (7.2)
**Race, n (%)**		
	White	517 (81)
	Black	63 (10)
	Other	57 (9)
**Children, n (%)**		
	0	367 (57)
	1	167 (26)
	≥2	33 (16)
Income^a^ (US $), mean (SD)		66,656 (33,356)
**Education^b^, n (%)**		
	High school or less	164 (26)
	2 or 4 years of college	263 (41)
	Postgraduate	203 (32)
	Prefer not to answer	7 (1)

^a^Income was collected as a categorical variable but treated as a continuous variable in analysis.

^b^In the United States, “High School” is a general education intended to be universal and to continue till the age of 18 years, followed by college and postgraduate training for some individuals. For reference, according to the most recently released educational data from the US Census bureau, 39% of the population aged ≥18 years had completed high school or had a lower education, 49% had completed some college but no postgraduate degree, and 12% had completed a postgraduate degree [23].

**Table 2:** Actions taken by study participants to decrease COVID-19 infection risk.

Self-reported protective actions			Participants, n (%)
**Most effective actions**			
	Washed hands with soap or used hand sanitizer several times per day	626 (98)	
	Wore a face mask	614 (97)	
	Avoided public spaces, gatherings, or crowds	525 (82)	
**Other effective actions**			
	Avoided contact with people who could be at high risk	503 (79)	
	Avoided eating at restaurants	479 (75)	
	Canceled or postponed personal or social activities	460 (72)	
	Worked or studied at home	345 (54)	
	Ordered meals or groceries to be delivered	340 (53)	
	Avoided your place of worship	285 (45)	
	Canceled or postponed work or school activities	243 (38)	
	Canceled or postponed air travel for pleasure	201 (32)	
	Canceled or postponed air travel for work	109 (17)	
**Indeterminate effectiveness actions**			
	Visited a doctor	270 (42)	
	Canceled a doctor’s appointment	97 (15)	
**Other unnecessary or ineffective actions**			
	Wiped down items from the grocery store	294 (46)	
	Wiped down packages with disinfectant	262 (41)	
	Stockpiled food or water	217 (34)	
	Took a hot bath	139 (22)	
	Ate garlic	84 (13)	
**Ineffective and potentially harmful actions**			
	Used a hand dryer instead of hand washing to kill the virus with heat	32 (5)	
	Rinsed nose with saline	22 (4)	
	Sprayed self with alcohol or chlorine	26 (4)	
	Used other medicines or supplements not prescribed by a doctor	17 (3)	
	Used an ultraviolet disinfection lamp	11 (2)	
	Used antibiotics	11 (2)	

### Information Source and Most Effective Actions

In regression analysis, those who were more likely to seek information from the CDC (*P*=.002), the WHO (*P*=.01), local health departments (*P*=.006), health care workers (*P*=.03), and public media (*P*=.04) practiced more of the 3 most effective protective actions (Table 3). Those who were less likely to obtain information from the US president (Donald Trump) or Vice-President (Mike Pence) at the time (*P*=.02) also practiced more of the most effective actions. The number of most effective actions engaged in was also positively associated with older age (*P*=.006) and negatively associated with later date of surveying (*P*=.003), higher number of children (*P*=.02), and the presence of COVID-19 symptoms (*P*=.04).

**Table 3:** COVID-19 information sources and most effective and potentially harmful actions.

News source				Respondents citing this source or trait, n (%)		Regression coefficient for the most effective actions (95% CI)		*P* value		Log odds ratio for potentially harmful actions (95% CI)		*P* value
Centers for Disease Control and Prevention				505 (79)		*0.18 (0.07 to 0.29)* ^a^		*.002*		–*0.82 (–1.52 to –0.11)*		*.02*
Local Department of Heath				425 (67)		*0.12 (0.04 to 0.21)*		*.006*		–0.17 (–0.76 to 0.41)		.57
World Health Organization				313 (49)		*0.11 (0.02 to 0.20)*		*.01*		0.29 (–0.31 to 0.90)		.34
US Department of Health				208 (33)		0.01 (–0.08 to 0.10)		.80		*0.74 (0.14 to 1.34)*		*.02*
President Donald Trump or Vice-President Mike Pence				75 (12)		–*0.16 (–0.29 to –0.03)*		*.02*		0.04 (–0.79 to 0.87)		.92
Health care workers				405 (64)		*0.09 (0.01 to 0.17)*		*.03*		*0.59 (0.03 to 1.16)*		*.04*
Friends and family				206 (32)		0.03 (–0.06 to 0.13)		.51		*1.04 (0.44 to 1.64)*		*.001*
Internet or social media				137 (22)		0.08 (–0.02 to 0.19)		.13		–0.17 (–0.86 to 0.51)		.62
Coworkers				123 (19)		0.01 (–0.09 to 0.12)		.82		0.06 (–0.62 to 0.74)		.86
Local news				192 (30)		0 (–0.1 to 0.09)		.92		0.57 (–0.03 to 1.16)		.06
Public media				161 (25)		*0.10 (0.00 to 0.19)*		*.04*		–0.63 (–1.34 to 0.03)		.06
National newspapers				120 (19)		0.04 (–0.07 to 0.15)		.45		–0.70 (–1.48 to 0.09)		.08
CNN				112 (18)		0.07 (–0.05 to 0.19)		.28		–0.2 (–1.03 to 0.62)		.63
NBC news				72 (11)		–0.04 (–0.2 to 0.11)		.60		–1.39 (–2.73 to 0.04)		.04
Fox News				65 (10)		0.01 (–0.14 to 0.16)		.87		0.76 (–0.13 to 1.65)		.09
ABC news				61 (10)		–0.05 (–0.23 to 0.13)		.57		–0.13 (–1.31 to 1.05)		.83
MSNBC				49 (8)		0.05 (–0.13 to 0.23)		.57		0.26 (–0.90 to 1.43)		.66
CBS news				52 (8)		–0.07 (–0.28 to 0.14)		.51		*1.48 (0.21 to 2.76)*		*.02*
**Other covariates**												
	Age (per 10 years)			N/A^b^		*0.08 (0.02 to 0.14)*		*.006*		0.24 (–0.17 to 0.66)		.25
	**Race**											
		White	N/A		Reference				Reference			
		Black	N/A		0.12 (–0.03 to 0.26)		.11		0.25 (–0.55 to 1.06)		.54	
		Other	N/A		0.11 (–0.02 to 0.24)		.09		*1.15 (0.40 to 1.90)*		*.003*	
	Income (per US $10,000)			N/A		–0.00 (–0.02 to 0.01)		.75		–0.04 (–0.14 to 0.06)		.40
	**Education**											
		High school or less	N/A		–0.07 (–0.20 to 0.07)		.33		0.16 (–0.69 to 1.01)		.72	
		Collegiate	N/A		0.02 (–0.08 to 0.11)		.73		0.39 (–0.27 to 1.04)		.25	
		Postgraduate	N/A		Reference				Reference			
		Prefer not to answer	N/A		–0.25 (–0.62 to 0.12)		.18		–0.30 (–2.82 to 2.23)		.82	
	Number of children			N/A		–*0.05 (–0.09 to –0.01)*		*.02*		–0.01 (–0.27 to 0.28)		.97
	COVID-19–related distress			193 (30)		0.01 (–0.08 to 0.09)		.87		–0.23 (–0.80 to 0.34)		.44
	COVID-19 symptoms			14 (2)		–*0.26 (–0.52 to –0.01)*		*.04*		–0.10 (–1.90 to 1.71)		.92
	High risk pregnancy history			276 (43)		0.01 (–0.07 to 0.09)		.78		–0.44 (–1.00 to 0.12)		.13
	Date of survey completion			N/A		–*0.026 (–0.04 to –0.01)*		*.003*		*0.15 (0.03 to 0.26)*		*.01*

^a^Italicized values are statistically significant. Regression coefficients for the most effective actions were generated through linear regression predicting each additional “most effective” action. Log-transformed odds ratios for harmful actions were generated through logistic regression analysis predicting *any* “misguided and potentially harmful” action. For “date of survey completion,” an increase in the regressor of 1 corresponds to a 30-day (1-month) change.

^b^N/A: not applicable.

### Information Source and Potentially Harmful Actions

Citing the following information sources was positively associated with engagement in any potentially harmful actions: the US Department of Health (*P*=.02), health care workers (*P*=.04), friends and family (*P*=.001), and CBS news (*P*=.02) (Table 3). Citing the CDC (*P*=.02) was negatively associated with engaging in harmful actions. Potentially harmful actions were positively associated with a later date (*P*=.01) and being of race other than White or Black. (*P*=.003).

### Most Effective Actions and Potentially Harmful Actions

On logistic regression analysis, the number of most effective actions engaged in was not associated with participation in any potentially harmful actions (*P*=.93) (Table 3).

Regression analyses showing associations between information sources and both “other effective” and “other unnecessary or ineffective” actions are shown in Multimedia Appendix 1.

## Discussion

### Principal Findings

In this local sample, pregnant people surveyed in the first 10 months of the COVID-19 pandemic in the United States reported multiple, varied COVID-19 information sources. These information sources were associated with individuals’ actions in several cases. Most significantly, we found that using the CDC as an information source was associated with most effective actions and negatively associated with harmful actions. The associations we found may have resulted from traits in the individuals we studied. For example, reporting the US president Donald Trump and Vice-President Mike Pence as an information source was associated with engaging in less protective actions, resonating with prior evidence that individuals’ political affiliations often influence their information source [24] and are associated with multiple COVID-19 protective actions [25-28]. Alternatively, sources may have been actively providing different information [14] or may have communicated similar information but with different levels of clarity or different degrees of targeting information specifically to pregnant audiences [29]. While trust in the CDC has repeatedly been shown to be associated with protective actions during the COVID-19 pandemic [13,30], other associations, including those of individual networks, are more difficult to explain. It is notable that professional pregnancy and maternal health organizations were not listed as sources in open-ended responses since these may be the most reliable sources of targeted up-to-date scientific information for this population.

Overall, we found that participation in the most effective protective actions was relatively high, with more than 90% of our pregnant sample reporting mask-wearing and frequent hand-washing. However, a nontrivial minority reported participating in at least one misguided or potentially harmful action, and we did not detect an association between participating in effective actions and abstaining from harmful actions. Trusted public health sources may need to directly address which actions are not helpful, particularly for populations for whom there may be additional uncertainty around the risk that is posed to them. Indeed, it is possible that an excess of fear and uncertainty, rather than lack of information, drives engagement in behaviors without an evidence base. This echoes a prior research finding that COVID-19 conspiracy theorists showed increased rates of protective behaviors, both those that were and those that were not recommended by governmental bodies [31].

In regression analysis controlling for information source, respondents’ reports of participation in effective actions were lower, and reports of participation in potentially harmful actions was higher, over the time course of the pandemic. This finding suggests that pregnant people, although often more likely than nonpregnant people to participate in evidence-based health behaviors [32,33], may have experienced flagging motivation to adhere to guidelines—that is, “pandemic fatigue” [34,35]—over time. Alternatively, given our findings suggesting the importance of information sources, it is also possible that as pregnant people encountered more sources of information and disinformation, they had decreasing clarity over which of their actions were evidence-based, leading to a perverse use of harmful actions in pursuit of greater protection. Our survey was launched early in the pandemic, when the pregnant population may have had to rely largely on their own “mental models” of safe behavior when engaging in proactive actions. It is possible that these intuitions about safe behavior, earlier during the pandemic, were clearer to pregnant people than the conflicting or unclear recommendations they may have received from other channels over the course of the survey time period.

To our knowledge, this is the first study to report that a decline in effective actions over the course of a pandemic may be associated with a rise in spurious or dangerous actions [12]. This finding requires confirmation, ideally through longitudinal surveys that can track individual rather than population-level changes in participation in recommended and spurious, potentially harmful actions over time during a public health emergency. While we would expect time-related behavioral changes attributable or related to pandemic fatigue to be replicated in other populations, at other times, and in other public health crises, this might not be true for changes related to unclear recommendations from information sources.

The data analyzed here, which were collected through a health app integrated into routine care, also demonstrates the potential role that health apps may play in alerting clinicians to health behaviors of patients at the individual or population level. We have previously reported how the MyHealthyPregnancy app collects user-reported risk information, such as violence toward intimate partners or drug adherence, directly providing resources and alerting clinicians when critical risks are identified [36,37]. Such tools could also serve as a platform to deliver responsive information campaigns to counter health misconceptions or misinformation.

### Limitations

This study has several limitations. These cross-sectional data can demonstrate associations between information sources and behaviors but cannot prove causality, and can determine changes within a population but not within individuals, as might be achieved through repeat sampling [34]. We focus here on a select population of pregnant people who engaged with a health-tracking app, which may limit the generalizability of our findings to other populations. In addition, the continuously changing dynamics of the COVID-19 pandemic were both informative and limiting. We were able to comment on changes in actions as the pandemic progressed. However, as the pandemic continues to evolve, information sources may shift owing to elections and changing media landscapes, and pregnant people will face new decisions around health behaviors as well.

### Conclusions

Pregnant people are now faced with the need to make decisions regarding COVID-19 vaccination and booster vaccination [38,39] and are adjusting their health behaviors as those around them are vaccinated. Pregnant people may further adjust their health behaviors in response to SARS-CoV-2 variants and other developments. As they continue to face additional contexts with uncertainty, dissemination of and adherence to health guidance will continue to be an important determinant of health at the population level. We found that our respondents accessed health information from several sources and that health behaviors may shift over time from effective to potentially harmful behaviors, regardless of information source. As pregnancy-relevant data continues to be gathered across agencies and institutions, it is critical that it be made widely publicly available and disseminated in accordance with best practices in health communication [14]. Most strikingly, perhaps, our findings show that even those individuals motivated to engage in “best-practice” behaviors were not necessarily less likely to also practice ineffective or harmful behaviors. Moving forward, key health organizations that are routinely viewed as sources of reliable health information, such as the CDC, should make a concerted effort to offer structured guidance on what *should* and *should not* be practiced in terms of protective behaviors for this population specifically.

## Appendix

Multimedia Appendix 1 COVID-19 information sources and other effective and other unnecessary or ineffective actions.

## Abbreviations

CDC: Centers for Disease Control and Prevention
MHP: MyHealthyPregnancy
WHO: World Health Organization
