# Supplementary material for: COVID-19 Information Sources and Health Behaviors During Pregnancy: Results From a Prenatal App-Embedded Survey
Source: JMIR Infodemiology. 2021 Dec 7;1(1):e31774. doi: 10.2196/31774 (PMC8664132; doi:10.2196/31774)
Supplement: Multimedia Appendix 1 [file infodemiology_v1i1e31774_app1.docx]

**Multimedia Appendix 1**: COVID-19 information sources and other effective and other unnecessary or ineffective actions

| **News Source** |  | **Regression coefficient for other effective actions (95% CI)** | ***P* value** | **Regression coefficient for other ineffective actions (95% CI)** | ***P* value** |
| --- | --- | --- | --- | --- | --- |
|  |  |  |  |  |  |
| CDC |  | 0.24 (-0.23, 0.72) | .31 | 0.08 (-0.21, 0.38) | .58 |
| Local DOH |  | **0.79 (0.42, 1.17)** | **<.001** | 0.05 (-0.18, 0.28) | .66 |
| WHO |  | **0.38 (0.00, 0.75)** | **.047** | **0.32 (0.09, 0.55)** | **.006** |
| US DOH |  | **0.38 (0.00, 0.76)** | .05 | 0.11 (-0.12, 0.34) | .35 |
| President Trump/VP Pence |  | -0.47 (-1.03, 0.09) | .10 | -0.1 (-0.45, 0.24) | .55 |
| Healthcare Workers |  | 0.34 (-0.01, 0.69) | .05 | **0.32 (0.11, 0.53)** | **.003** |
| Friends and family |  | 0.34 (-0.07, 0.74) | .11 | **0.25 (0.00, 0.5)** | **.05** |
| Internet/Social Media |  | -0.11 (-0.57, 0.34) | .63 | 0.06 (-0.22, 0.34) | .69 |
| Coworkers |  | -0.03 (-0.49, 0.42) | .89 | -0.12 (-0.4, 0.16) | .39 |
| Local News |  | 0.17 (-0.22, 0.57) | .39 | 0.14 (-0.11, 0.38) | .27 |
| Public media |  | -0.02 (-0.42, 0.38) | .92 | 0.12 (-0.13, 0.37) | .34 |
| National Newspapers |  | **0.50 (0.04, 0.96)** | **.03** | -0.16 (-0.44, 0.12) | .26 |
| CNN |  | **0.53 (0.02, 1.05)** | **.04** | 0.29 (-0.02, 0.61) | .07 |
| NBC news |  | -0.58 (-1.24, 0.09) | .09 | -0.11 (-0.52, 0.3) | .60 |
| Fox News |  | 0.45 (-0.18, 1.09) | .16 | 0.24 (-0.15, 0.63) | .23 |
| ABC news |  | **-0.78 (-1.54, -0.02)** | **.05** | -0.32 (-0.79, 0.15) | .18 |
| MSNBC |  | 0.20 (-0.57, 0.96) | .61 | -0.1 (-0.57, 0.37) | .68 |
| CBS news |  | 0.03 (-0.88, 0.94) | .95 | 0.23 (-0.33, 0.79) | .42 |
| **Other covariates** |  |  |  |  |  |
| Age (per 10 years) |  | 0.21 (-0.03, 0.45) | .09 | 0.08 (-0.07, 0.23) | .27 |
| Race |  |  |  |  |  |
|  | White | reference |  | reference |  |
|  | Black | **0.62 (0.01, 1.23)** | **.05** | **0.51 (0.13, 0.89)** | **.008** |
|  | Other | **0.71 (0.15, 1.27)** | **.01** | **0.43 (0.09, 0.77)** | **.01** |
| Income (per $10,000) |  | **0.09 (0.02, 0.16)** | **.01** | **-0.04 (-0.08, 0)** | **.05** |
| Education |  |  |  |  |  |
|  | High school or less | **-1.15 (-1.71, -0.59)** | **<.001** | -0.22 (-0.56, 0.13) | .22 |
|  | Collegiate | **-0.31 (-.0.70, 0.08)** | **.12** | -0.15 (-0.39, 0.09) | .23 |
|  | Postgraduate | reference |  | reference |  |
|  | Prefer not to answer | **-2.85 (-4.43, -1.28)** | <.001 | -0.39 (-1.36, 0.58) | .44 |
| Number of children |  | 0.05 (-0.12, 0.23) | .56 | 0.02 (-0.09, 0.13) | .77 |
| COVID-19-related distress |  | **0.45 (0.09, 0.82)** | **.02** | 0.09 (-0.13, 0.32) | .41 |
| COVID-19 symptoms |  | 0.15 (-0.95, 1.24) | .80 | 0.23 (-0.45, 0.9) | .51 |
| High risk pregnancy history |  | 0.11 (-0.23, 0.46) | .52 | 0.04 (-0.17, 0.26) | .69 |
| Date of survey completion |  | **-0.23 (-0.3, -0.15)** | **<.001** | **-0.13 (-0.17, -0.08)** | **<.001** |

Regression Coefficients were generated in linear regressions predicting each additional “other effective” or “other ineffective” action. *CDC* Centers for Disease Control and Prevention; *Local DOH* Local Departments of Health; *US DOH* United States Department of Health (These were asked individually because departments at the state or county level may have policies and messaging differing from the US DOH); *WHO* World Health Organization. Please note that for “date of survey completion”, an increase in the regressor of 1 corresponds to a 30 day (1-month) change.
